# Supplementary figures and images for: Herpes Simplex Virus Type-2 Paralyzes the Function of Monocyte-Derived Dendritic Cells
Source: Viruses. 2020 Jan 16;12(1):112. doi: 10.3390/v12010112 (PMC7019625; doi:10.3390/v12010112)

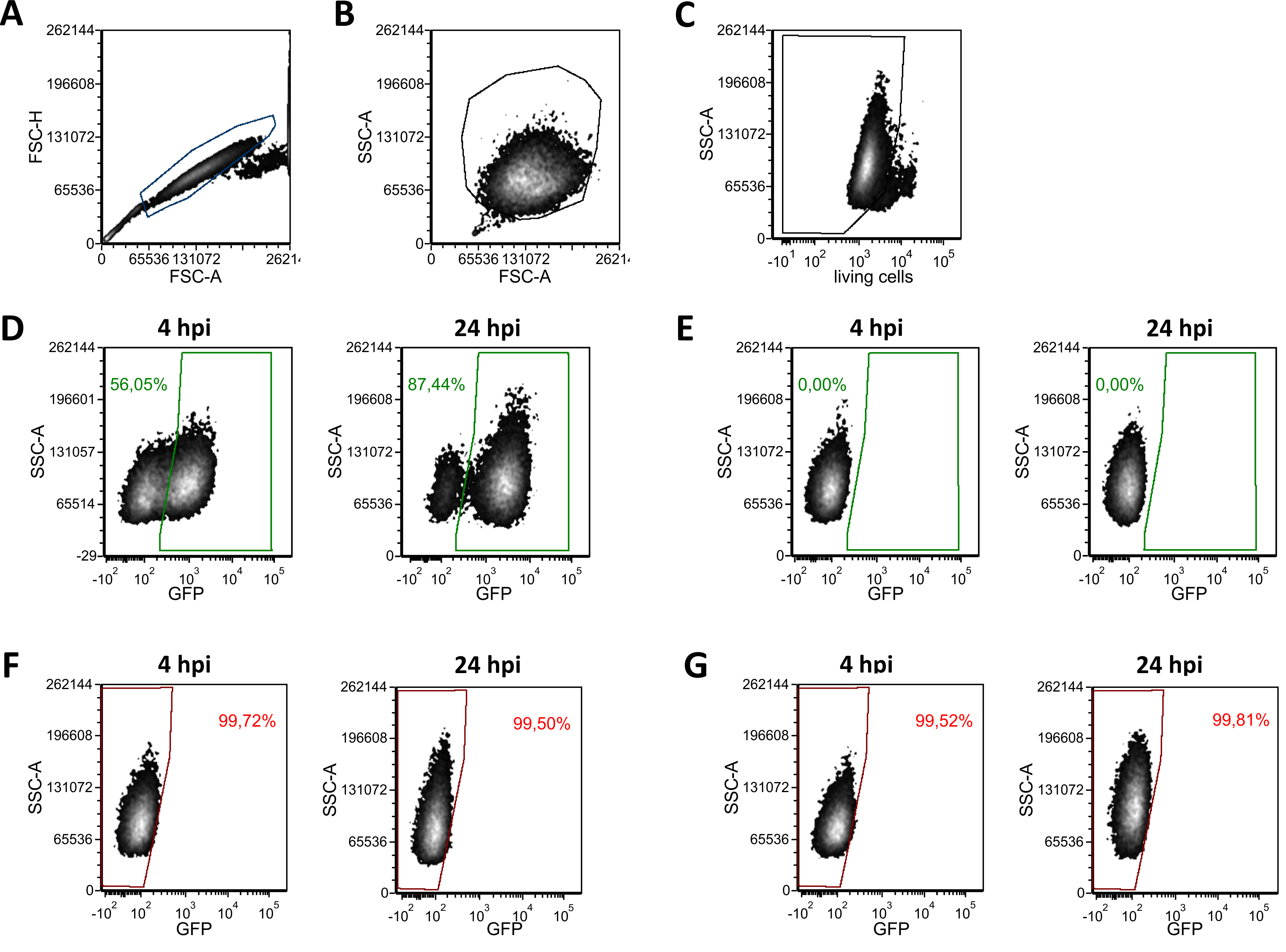

Supplement: Supplementary file 1 [file viruses-12-00112-s001.zip › Supplement Figures/Suppl Figure 1.tif]

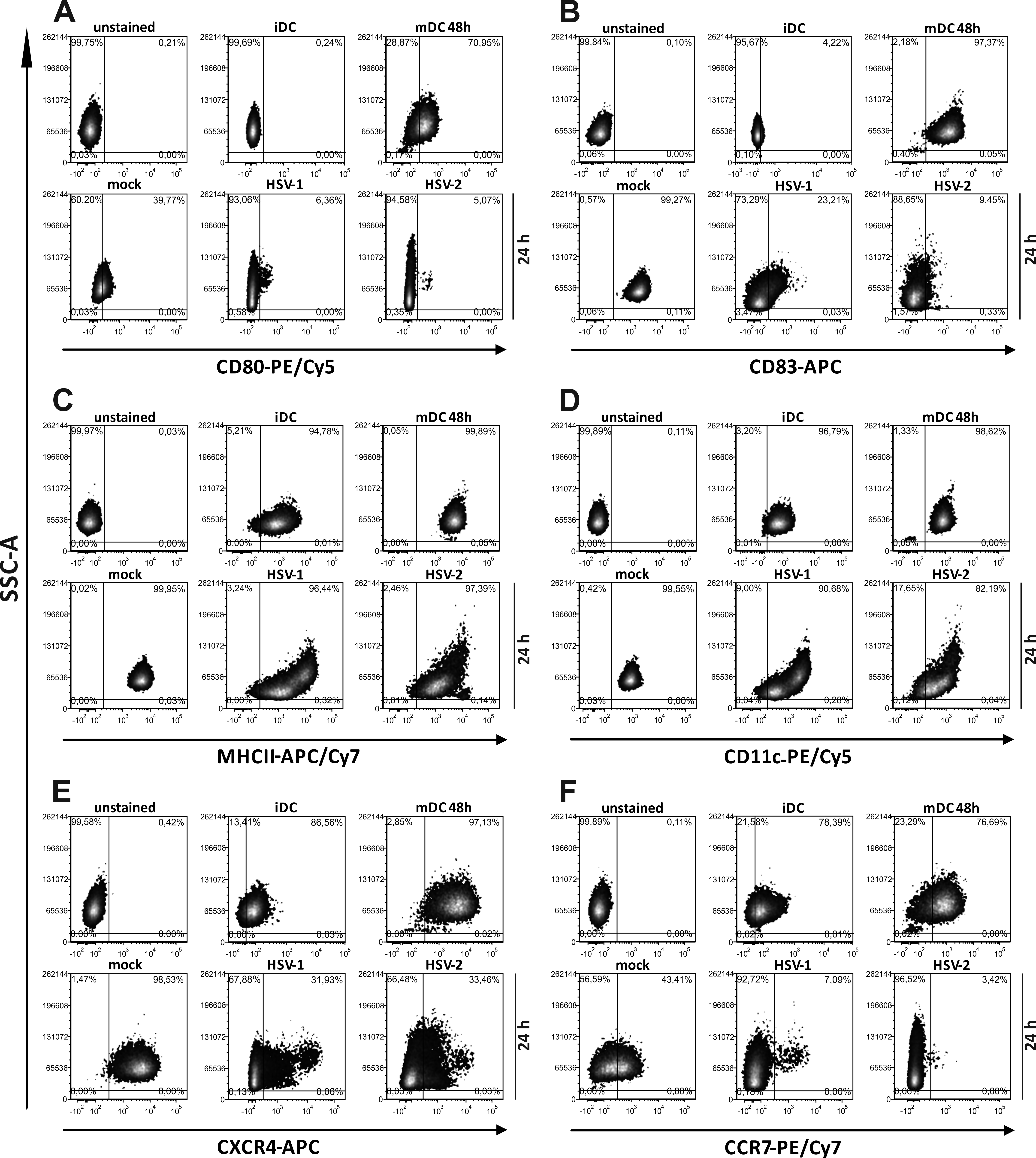

Supplement: Supplementary file 1 [file viruses-12-00112-s001.zip › Supplement Figures/Suppl Figure 2.tif]

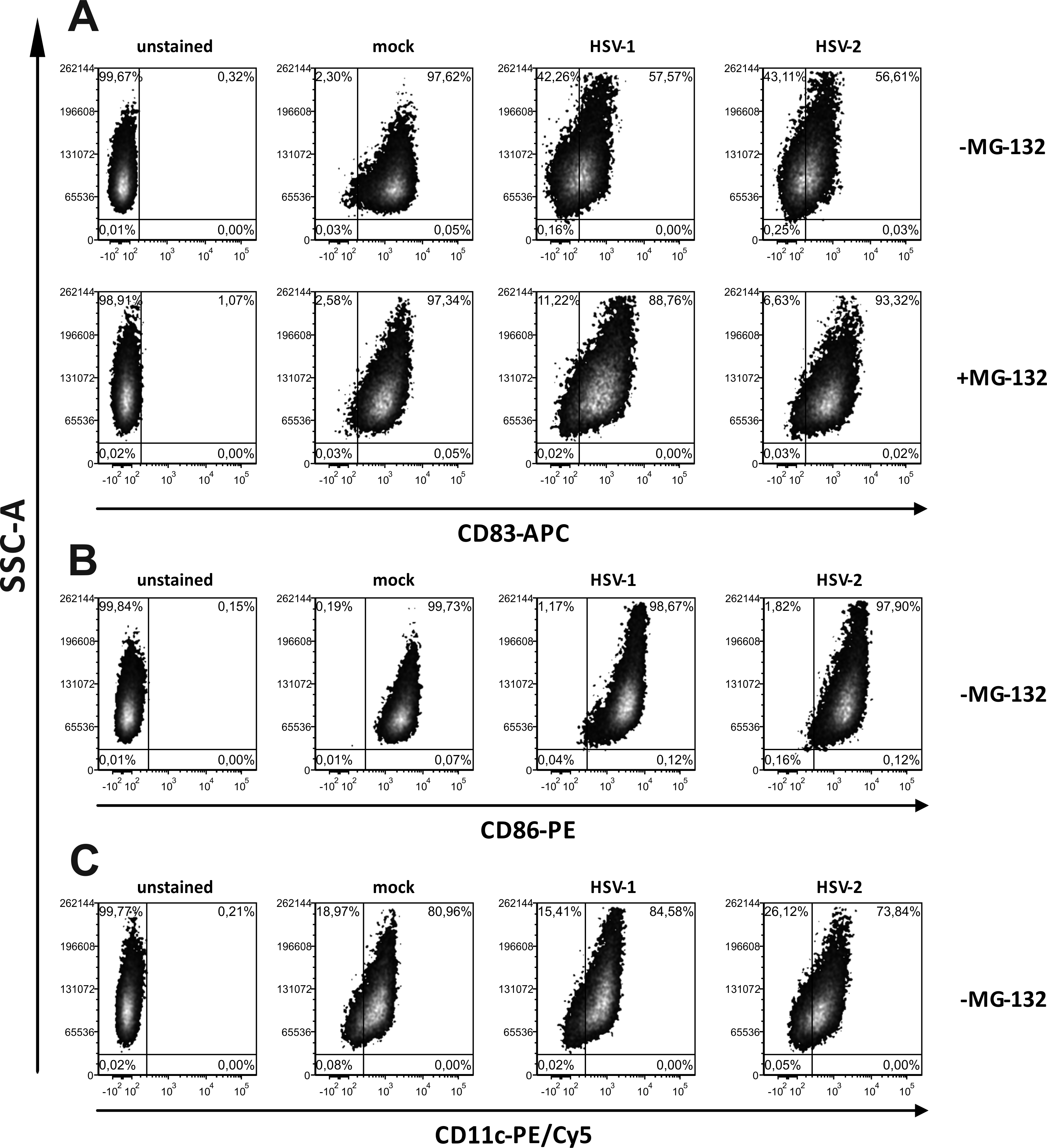

Supplement: Supplementary file 1 [file viruses-12-00112-s001.zip › Supplement Figures/Suppl Figure 3.tif]

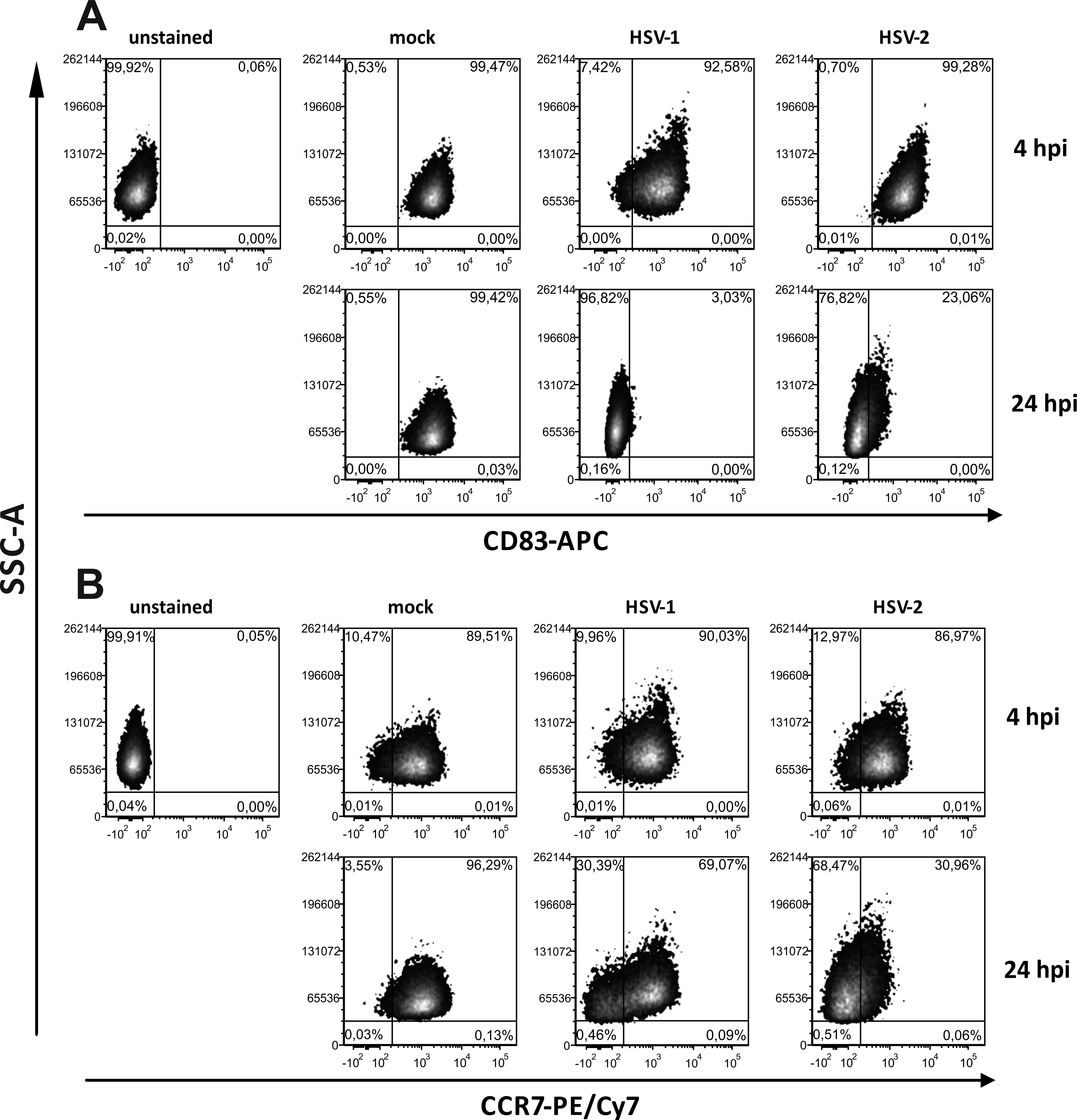

Supplement: Supplementary file 1 [file viruses-12-00112-s001.zip › Supplement Figures/Suppl Figure 4.tif]

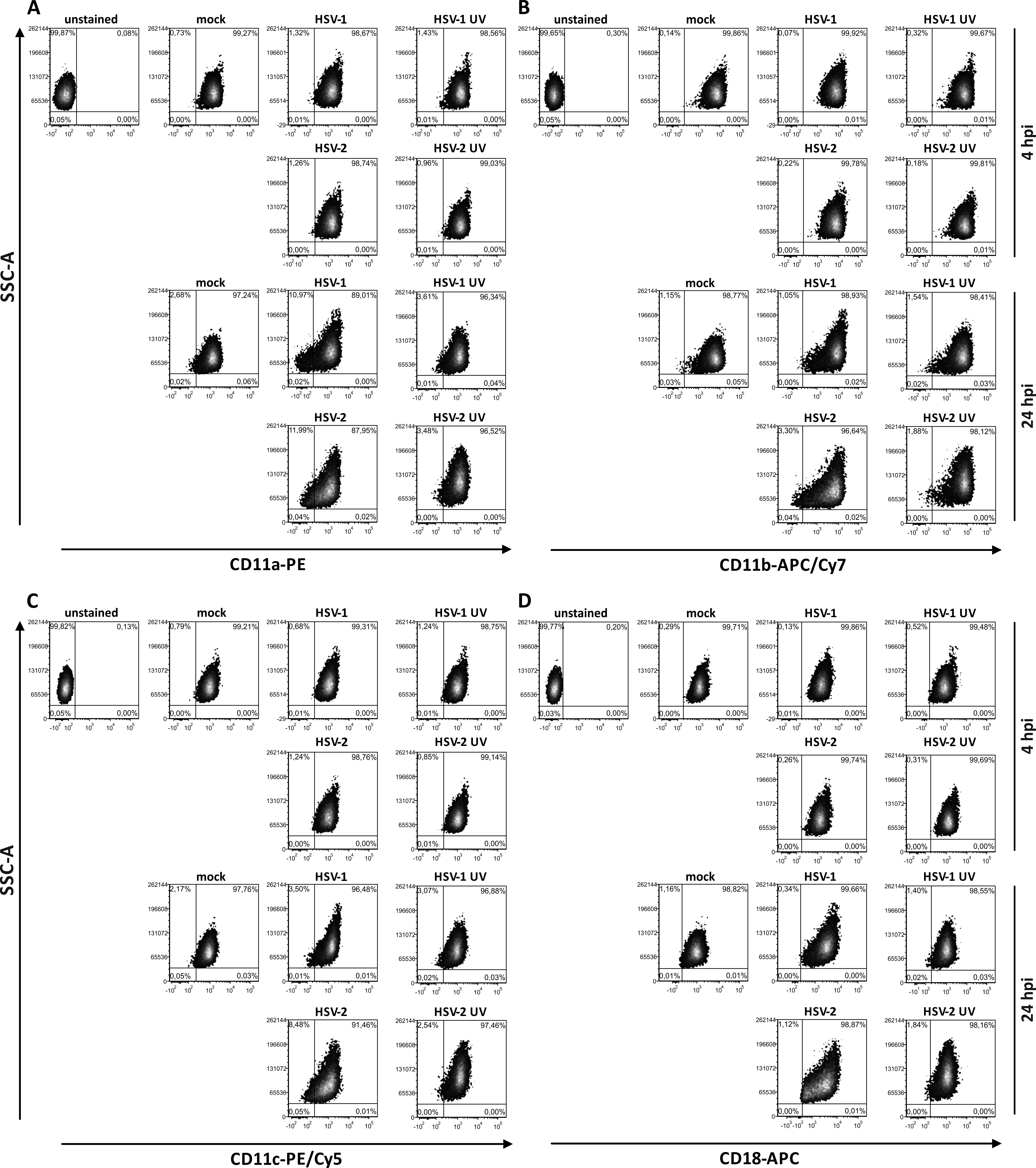

Supplement: Supplementary file 1 [file viruses-12-00112-s001.zip › Supplement Figures/Suppl Figure 5.tif]

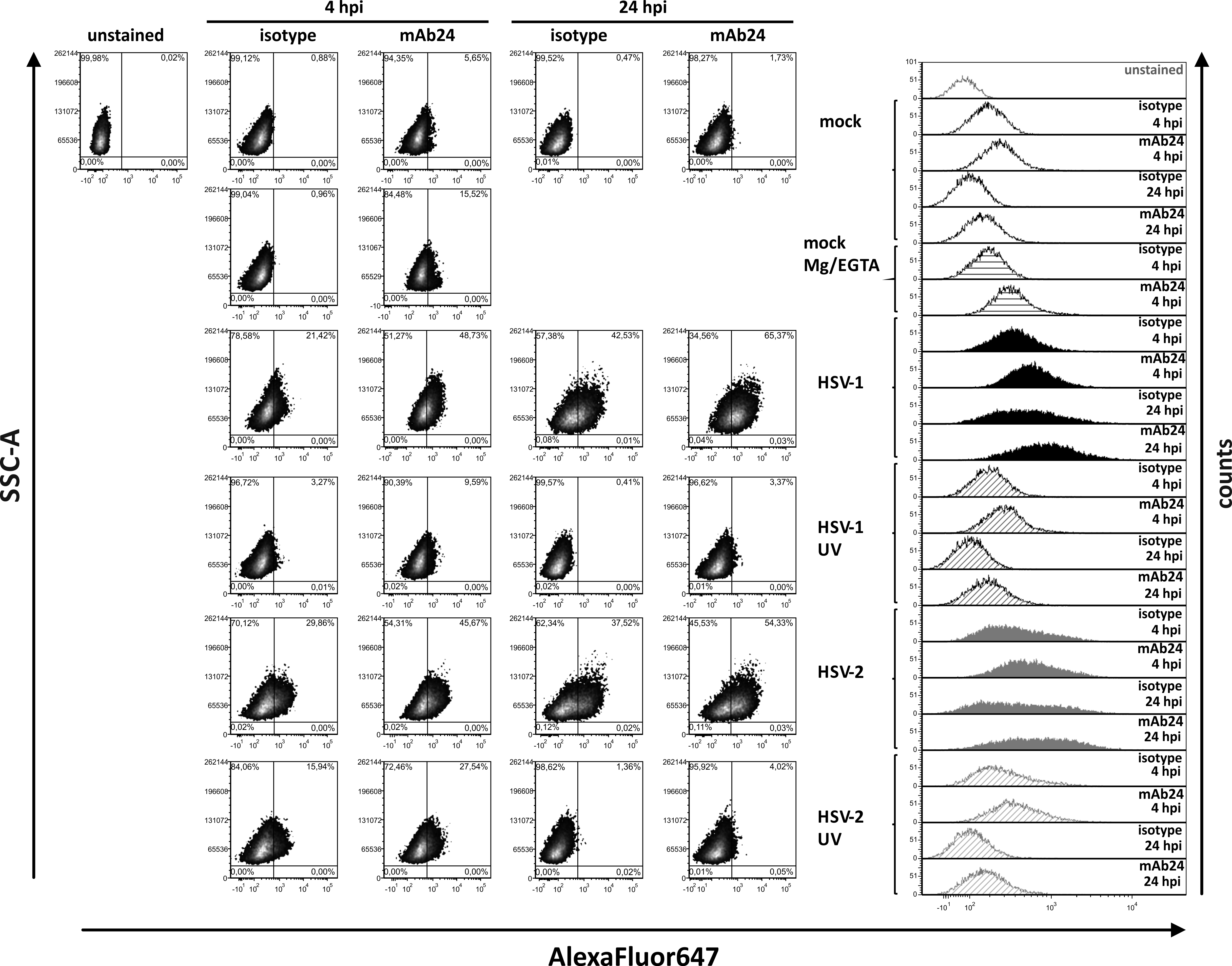

Supplement: Supplementary file 1 [file viruses-12-00112-s001.zip › Supplement Figures/Suppl Figure 6.tif]
